# Re-engineering an American emergency department with Team Triage - adapting to increasing patient volume in emergency services

**DOI:** 10.1186/1753-6561-6-S4-P52

**Published:** 2012-07-09

**Authors:** L Haruno, O Geling, J Kakuda

**Affiliations:** 1 University of Notre Dame, USA/Hawaii Pacific Health, USA; 2 University of Hawaii/Hawaii Pacific Health, USA; 3 Pali Momi Medical Center/Hawaii Pacific Health, USA

## Introduction

Overcrowding in U.S. emergency departments (ED) is a critical issue. In a privatized health care system, patients with limited access to insurance benefits (as influenced by challenging socio-economic conditions) often utilize the ED as crucial point of access to care. Non-acute patients seeking primary care in ED facilities can congest operations and contribute to overcrowding. In Hawaii’s second busiest ED (Hospital 1), a non-traditional method of emergency triage – team triage – has been implemented to improve patient throughput and satisfaction, and mitigate effects of overcrowding. This study defines trends and compares two prominent and nationally recognized EDs (Hospital 1 and Hospital 2) in the state of Hawaii - employing team and traditional triage respectively, to examine changes in patient population, demographics, acuteness, and departmental throughput measures.

## Methods

Retrospective data review from 180,000+ patient records (112,000+ from Hospital 1, and 67,000+ from Hospital 2) was obtained from July 2007 – June 2010 through the electronic medical records system, EPIC. Data included patient demographics, mode of arrival, length of stay (LOS), door-to-doctor time, insurance type, and patient satisfaction. Statistical analysis was completed with JMP and SPSS statistical software.

## Results

Patient volume increased over the 36 month time period at both hospitals as did patient satisfaction, particularly after the implementation of team triage at Hospital 1. The door-to-doctor time decreased for outpatients at both hospitals as well, but the overall LOS for outpatients remained largely the same. The percentage of governmentally insured (as opposed to privately insured) patients increased, but surrogate measures of acuteness - ambulance arrivals and admission rates – declined amongst this population.

## Conclusions

Trends in satisfaction and door-to-doctor time are not exclusive to a team triage environment. Team triage potentially reduces door-to-doctor time, but has minimal impact on overall LOS. There exists a disparity in patient population and relative acuity that can contribute to overcrowding and patient throughput. Ultimately inpatient resources – not method of triage or even ED throughput – may be the biggest determining factor in improving overcrowding in EDs outside of health policy changes and reform.

